# Graft flow assessment and early coronary artery bypass graft failure: a computed tomography analysis

**DOI:** 10.1093/icvts/ivab298

**Published:** 2021-10-27

**Authors:** Andrea D’Alessio, Ioannis Akoumianakis, Andrew Kelion, Dimitrios Terentes-Printzios, Andrew Lucking, Sheena Thomas, Danilo Verdichizzo, Amar Keiralla, Charalambos Antoniades, George Krasopoulos

**Affiliations:** 1 Department of Cardiothoracic Surgery, Oxford University Hospital NHS Foundation Trust, Oxford, UK; 2 Cardiovascular Medicine Division, University of Oxford, Oxford, UK; 3 Department of Cardiology, Oxford University Hospitals NHS Foundation Trust, Oxford, UK; 4 Department of Cardiac Anesthesia, Oxford University Hospitals NHS Foundation Trust, Oxford, UK

**Keywords:** Coronary artery bypass graft, Graft failure, Intraoperative graft flow, Transit time flow measurement, Computed tomography angiography, Endoscopic vein harvesting

## Abstract

**OBJECTIVES:**

We evaluated graft patency by computed tomography and explored the determinants of intraoperative mean graft flow (MGF) and its contribution to predict early graft occlusion.

**METHODS:**

One hundred and forty-eight patients under a single surgeon were prospectively enrolled. Arterial and endoscopically harvested venous conduits were used. Intraoperative graft characteristics and flows were collected. Graft patency was blindly evaluated by a follow-up computed tomography at 11.4 weeks (median).

**RESULTS:**

Graft occlusion rate was 5.2% (*n* = 22 of 422; 8% venous and 3% arterial). Thirteen were performed on non-significant proximal stenosis while 9 on occluded or >70% stenosed arteries. Arterial and venous graft MGF were lower in females (*P*_arterial_ = 0.010, *P*_venous_ = 0.009), with median differences of 10 and 13.5 ml/min, respectively. Arterial and venous MGF were associated positively with target vessel diameter ≥1.75 mm (*P*_arterial_ = 0.025; *P*_venous_ = 0.002) and negatively with pulsatility index (*P*_arterial_ < 0.001; *P*_venous_ < 0.001). MGF was an independent predictor of graft occlusion, adjusting for EuroSCORE-II, pulsatility index, graft size and graft type (arterial/venous). An MGF cut-off of 26.5 ml/min for arterial (sensitivity 83.3%, specificity 80%) and 36.5 ml/min for venous grafts (sensitivity 75%, specificity 62%) performed well in predicting early graft occlusion.

**CONCLUSIONS:**

We demonstrate that MGF absolute values are influenced by coronary size, gender and graft type. Intraoperative MGF of >26.5 ml/min for arterial and >36.5 ml/min for venous grafts is the most reliable independent predictor of early graft patency. Modern-era coronary artery bypass graft is associated with low early graft failure rates when transit time flow measurement is used to provide effective intraoperative quality assurance.

**Figure:**
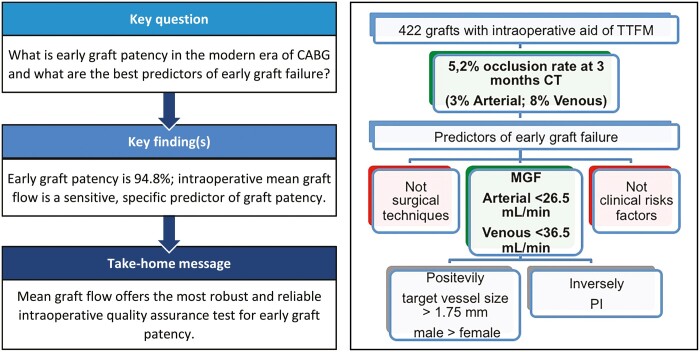

## INTRODUCTION

Coronary artery bypass graft (CABG) surgery is considered the gold standard for the treatment of patients with advanced coronary artery disease, with randomized clinical trials corroborating its effectiveness [1]. Despite low postoperative complication rates and contemporary CABG techniques [2], early graft failure remains an important concern. Several technical aspects also remain controversial, including the use of openly versus endoscopically harvested grafts [3], routine use of arterial grafts [4, 5] and systematic preoperative assessment of proximal target vessel stenosis [6]. Therefore, there is an unmet need for a more standardized operative strategy and a better understanding of the predictors of graft failure, in order to optimize patency and expand our capacity to deliver adequate CABG grafts [7].

Intraoperative assessment of graft flow is believed to be a useful index of graft quality, which may be associated with short- and mid-term graft failure [8].

In this study, we first evaluated potential determinants of intraoperative mean graft flow (MGF) and secondly examined the ability of MGF to predict early graft failure, as assessed by computed tomography (CT)-angiography.

## MATERIALS AND METHODS

This prospective, observational, non-blinded, non-randomized study included 148 consecutively enrolled patients, operated by a single surgeon. All patients had CABG performed and agreed to have postoperative CT-angiography.

### Ethics statement

Study protocols were in agreement with the declaration of Helsinki and all participants provided written informed consent prior to enrolment. Ethics approval was obtained from South-Central, Oxford C Research Ethics Committee, reference MREC 11/SC/0140.

### Methods

Data were prospectively collected and retrospectively analysed. Exclusion criteria included active inflammatory disease, neoplastic, stage-4 renal impairment (eGFR < 30 ml/min) or end-stage liver disease. Demographic characteristics and past medical history were obtained from patients’ notes and from direct interview of the patients by a research investigator (Table 1).

**Table 1:** Demographic characteristics of study participants

Participant number	148
Age	66.2 ± 11.0
Male sex (%)	131 (89)
Hypertension (%)	130 (88)
Hyperlipidaemia (%)	137 (93)
Diabetes mellitus (%)	31 (21)
CCS score	
0 (%)	3 (2)
1 (%)	6 (4)
2 (%)	32 (22)
3 (%)	95 (64)
4 (%)	12 (8)
NYHA class	
1 (%)	65 (44)
2 (%)	62 (42)
3 (%)	15 (10)
4 (%)	6 (4)
Previous AMI (%)	78 (53)
Smoking	
Never (%)	67 (45)
Ex (%)	77 (52)
Active (%)	4 (3)
PVD (%)	33 (22)
EF	
>50% (%)	130 (88)
30–50% (%)	18 (12)
On-pump surgery (%)	109 (74)
Associated valve surgery (%)	27 (18)
Mean graft per patient	2.85 ± 0.67

Continuous variables are presented as mean ± standard deviation.

AMI: acute myocardial infarction; CCS: Canadian Cardiovascular Society; EF: ejection fraction; NYHA: New York Heart Association; PVD: peripheral vascular disease.

All patients underwent CABG by a single surgeon experienced in both, on- and off-pump techniques. The majority of patients (74%) had their operation on-pump and 18% underwent concomitant valve surgery. Skeletonized internal mammary arteries (IMAs), endoscopically harvested saphenous veins or radial arteries were used as conduits, patients with sequential or Y-grafts were excluded from this cohort. Detailed surgical procedures are presented in the Supplementary Materials and Methods. Transit time flow measurement (TTFM) obtained with VeriQ^™^ (Medistim, Oslo, Norway) was used to assess the quality of the grafts during the operation. MGFs and pulsatility indexes (PIs) were recorded using a standardized technique, after protamine administration and with a mean systemic blood pressure between 70 and 80 mmHg. The patency of all grafts was assessed by coronary CT-angiography at a median of 11.4 weeks (range: 6–52 weeks) following CABG [9]. A graft was considered occluded when there was no opacification. The preoperative angiograms of all occluded grafts were retrospectively assessed using QAngio XA 3-dimensional software package (Medis Medical Imaging System, Leiden, Netherlands) and the grafted, native vessel’s diameter and degree of proximal stenosis (diameter and area) were measured [10]. The distal myocardial collateral bed filling was evaluated for all chronic total occlusion (CTO) arteries with occluded grafts, using standardized techniques [11, 12] (Supplementary Materials and Methods).

### Statistical analysis

Continuous variables were tested for normality using the Shapiro–Wilk test, and non-normally distributed variables were analysed with non-parametric tests or log-transformed prior to analysis. Univariate associations between continuous normally distributed variables were evaluated with Pearson’s correlation coefficient; continuous variable differences between 2 groups were assessed by Mann–Whitney *U*-tests or Student’s *t*-tests for independent (unpaired) measurements as appropriate. We estimated that 148 patients would allow for detecting an occlusion odds ratio of 0.49 per unit of the continuous predictor MGF, assuming a normal distribution of MGF, with the power of 0.9 and *α* = 0.05.

A mixed-effects multilevel multiple linear regression analysis was performed, where MGF was used as the dependent variable of interest whilst sex, graft type (arterial or venous), PI and target vessel size were used as independent covariates (those were the demographic- or graft-associated variables showing a trend for significant association with flow upon univariate analysis). To provide more insight into the differences between arterial and venous grafts, a secondary multiple linear regression sub-analyses were performed. Continuous variables were log-transformed. Standardized Beta-coefficients (*B*_stand._) are presented for each covariate.

Regarding the ability of MGF to predict graft occlusion, a mixed-effects, multilevel binary logistic regression analysis model was used to account for within-patients graft clustering. MGF was the principal independent variable of interest, adjusted for EuroSCORE-II, PI and graft type (dichotomized as arterial versus venous). Early graft failure was the dichotomous outcome of interest. Continuous variables were log-transformed. We maintained the continuous nature of continuous variables in our principal analysis, in order to maximize the data information towards addressing our primary statistical question, especially given the moderate sample size (hence odds ratios for continuous variables were defined per unit of each variable). Given the clinical necessity of using cut-off values to robustly select high- versus low-risk grafts, we also conducted a supplementary logistic regression sub-analyses per graft type, by using MGF as a dichotomous variable [with cut-off values determined by receiver operative characteristic analysis for arterial and venous grafts], with odds ratio being determined per group (high versus low MGF). There were no data censoring as this was not a time-to-event analysis and all cases were evaluated regarding the outcome.

All statistical analyses were performed using the SPSS statistical software version 24 (IBM Corp. Released 2016. IBM SPSS Statistics for Windows, Version 24.0, Armonk, NY, USA: IBM Corp). Two-tailed *P*-values were calculated and *P* < 0.05 was considered statistically significant. Robust variances were used in the regression models to accommodate possible assumption model violations.

## RESULTS

### Patients and graft descriptive data

A total of 422 grafts (235 arterial, 187 venous) were performed in 148 participants (121 CABG and 27 CABG + Valve). There was no intraoperative mortality, periprocedural myocardial infarctions, early recurrent angina, acute heart failure or early postoperative need for ercutaneous coronary intervention or graft revision. Overall early occlusion rate was 5.2% (*n* = 22), 8% (*n* = 15) of endoscopically harvested venous and 3% (*n* = 7) of arterial conduits (Supplementary Material, Table S1). Grafts to the intermediate, posterior descending and right coronary arteries resulted in higher incidence of occlusion rates (Supplementary Material, Table S2).

Subgroup analysis of the 22 occluded grafts using postoperative CT-angiography has demonstrated that 12 (55%) anastomosed onto native coronary arteries with non-significant narrowing of <70% (proximal lesion overestimated during the preoperative coronary angiography assessment), 6 (27%) were anastomosed onto totally occluded native coronary arteries (CTO) and 4 grafts (18%) had no clear cause that could contribute or justify their early occlusion.

Retrospective 3D-QCA analysis of the preoperative coronary angiogram for the same cohort of occluded grafts showed that 4 of the grafted native coronary arteries had significant proximal stenosis (18%—3 venous; 1 arterial), 13 had <70% stenosis (59%—6 arterial; 7 venous) and 5 vessels were CTOs (23%—5 venous; Supplementary Material, Table S3). All of the CTOs had medium myocardial bed and good collaterals with Grade 2 or 3 (Supplementary Material, Table S4).

The assessment of the proximal stenosis was similar between preoperative 3D-QCA and postoperative CT-angiography. The discrepancies were assigned to disease progression (1 case from <70% to >70% stenosis and 1 case from sub-occlusion to CTO).

### Determinants of intraoperative graft flow

**Figure 1:**
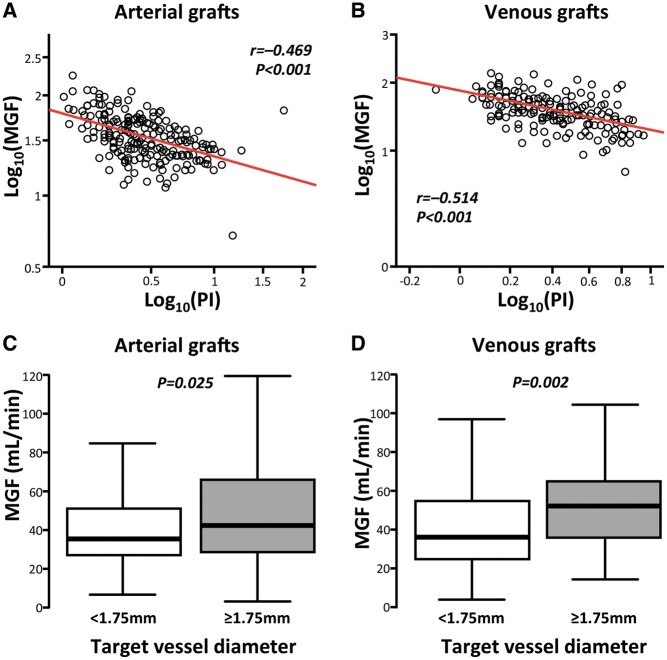
Surgical determinants of graft flow. MGF of both arterial and venous grafts was significantly and negatively correlated with PI, (**A** and **B**). A target vessel size ≥1.75 cm was associated with the increased flow for both arterial (**C**) and venous (**D**) grafts. *P*-values and Pearson *r* correlation coefficients are presented in (**A**) and (**B**). *P*-values in (**C**) and (**D**) are calculated by unpaired *t*-tests. MGF: mean graft flow; PI: pulsatility index.

Univariate analysis of demographic factors provided no evidence of association between MGF and age (*r*_arterial_ = –0.061, *P* = 0.51; *r*_venous_ = 0.061, *P* = 0.51), hyperlipidaemia (Mann–Whitney *U*; *P*_arterial_ = 0.70, *P*_venous_ = 0.81), diabetes (*t*-test; *P*_arterial_ = 0.75, *P*_venous_ = 0.85) or smoking status (Kruskal–Wallis; *P*_arterial_ = 0.31, *P*_venous_ = 0.08). Flow measurements were lower in females, with a median difference of 10 ml/min for arterial (*P* = 0.01) and 13.5 ml/min for venous grafts (*P* = 0.009; Supplementary Material, Fig. S1A and B). The same analysis has shown no evidence of association between MGF and the use of cardiopulmonary bypass (*t*-test_arterial_ = 0.50, *P*_venous_ = 0.38). MGF was, however, inversely correlated with PI (Fig. 1A and B) for both arterial and venous grafts and was also significantly and positively associated with the size of the target vessel (median ≥1.75 mm, Fig. 1C and D).

Multilevel multiple linear regression analysis integrating the significant demographic and surgical factors showed that PI alone was independently associated with MGF (*B*_stand_ = -0.536 and *P*_PI_ < 0.001; Table 2). Sub-analysis per graft type revealed that PI (*B*_stand_ = -0.216, *P*_PI_ = 0.014) and target vessel size (*B*_stand_ = 0.336, *P*_tvs_ < 0.001) were independently associated with MGF for arterial grafts, whilst venous graft MGF was only independently associated with PI (*B*_stand_ = -0.525, *P*_PI_ < 0.001; Table 3).

**Table 2:** Mixed-effects multiple linear regression model of mean graft flow determinants

Variable	*B*_stand._	Adjusted *P*-value
***PI***	−0.536*	<0.001*
Target vessel size	0.393	0.055
Graft type (arterial versus venous)	−0.030	0.89
Sex	−0.121	0.57

PI and target vessel size were log_10_-transformed prior to the analysis. Sex and graft type were inputted as dichotomous variables. * and bold italics are used to visually highlight the statistically significant results.

*B*_stand._; Standardized beta coefficient; PI: pulsatility index.

**Table 3:** Multiple regression sub-analyses of mean graft flow determinants per graft type

Variable	*B*_stand._	Adjusted *P*-value
Arterial grafts		
***PI***	−0.216*	0.014*
***Target vessel size***	0.336*	<0.001*
Sex	0.097	0.27
Venous grafts		
***PI***	−0.525*	<0.001*
Target vessel size	0.03	0.73
Sex	0.138	0.094

PI and target vessel size were log_10_-transformed prior to the analysis. Sex and graft type were inputted as dichotomous variables. * and bold italics are used to visually highlight the statistically significant results.

*B*_stand._; Standardized beta coefficient; PI: pulsatility index.

### Intraoperative mean graft flow and prediction of early graft failure

**Figure 2:**
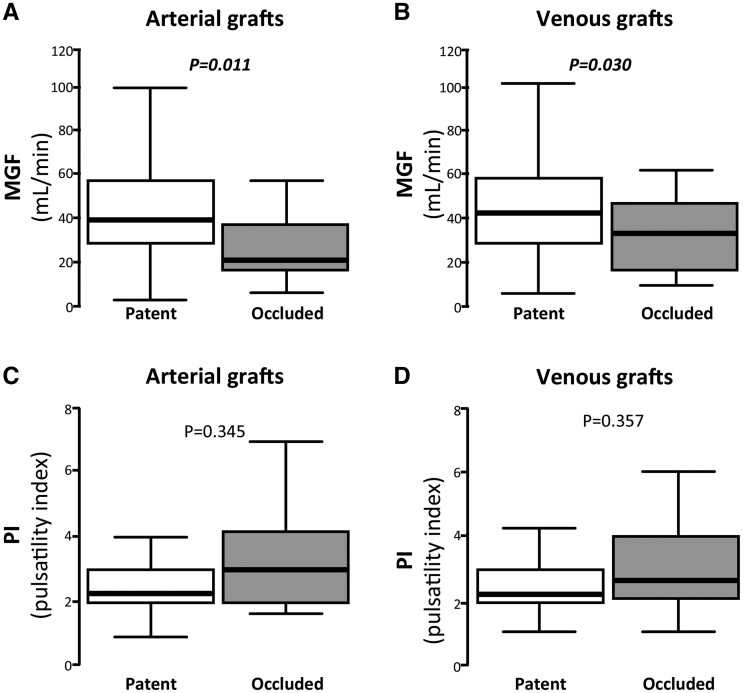
Graft occlusion and transit-time flow measurement parameters. MGF was reduced in arterial (**A**) and venous (**B**) occluded grafts. The association of occluded grafts with PI was non-significant (**C** and **D**). *P*-values are calculated by Mann–Whitney *U*-tests. MGF: mean graft flow; PI: pulsatility index.

Occluded arterial and venous grafts had significantly lower MGF (*P*_arterial_ = 0.011 *P*_venous_ = 0.030) without any significant association with PI (Fig. 2). Multilevel multiple binary logistic regression analysis revealed that MGF was independently associated with increased risk for early occlusion prediction, after adjusting for EuroSCORE-II, PI, target vessel size, graft type (arterial-v-venous) and accounting for within-patient clustering (Table 4).

**Table 4:** The role of mean graft flow in predicting early graft occlusion—mixed-effects binary logistic regression model

Variable	OR [95% CI]	Adjusted *P*-value
***MGF***	0.007 [0.003–0.303]*	0.010*
PI	0.66 [0.014–31.9]	0.83
Target vessel size	0.337 [0.010–83.8]	0.78
EuroSCORE-II	1.02 [0.190–5.5]	0.98
Graft type (venous versus arterial)	1.60 [0.130–19.9]	0.71

Graft type was inputted as a dichotomous variable. The rest of the variables were continuous and log_10_-transformed prior to the analysis, hence OR corresponds to a change by a factor 10. * and bold italics are used to visually highlight the statistically significant results.

MGF: mean graft flow, PI: pulsatility index.

**Figure 3:**
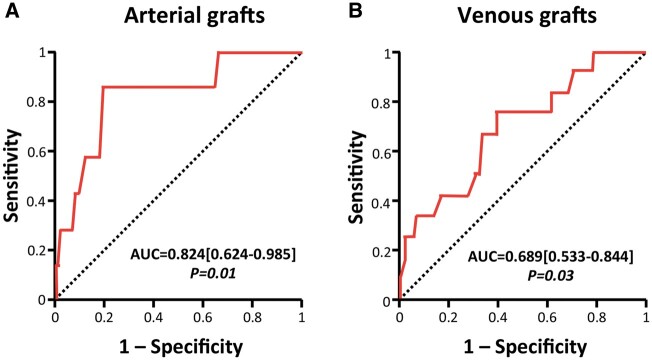
Receiver operating characteristic curve analysis for mean graft flow as an occlusion predictor. Receiver operating characteristic analysis was performed independently for arterial (**A**) and venous (**B**) grafts to interpolate the optimal cut-off value for mean graft flow. The AUC is presented for each curve. AUCs [95% confidence interval] and corresponding receiver operating characteristic P-values are presented for each model. AUC: area under the curve.

Receiver operative characteristic analyses with arterial and venous MGF showed that an MGF of <26.5 ml/min predicted early graft failure for arterial grafts with sensitivity 83.3% and specificity 80%, while MGF of <36.5 ml/min predicted early graft failure for venous grafts with sensitivity 75% and specificity 62% (Fig. 3A and B). Multiple logistic regression sub-analyses suggested that those cut-off values independently predicted arterial and venous occlusion risk, respectively, after adjusting for EuroSCORE-II, PI and target vessel size (Table 5).

**Table 5:** Sub-analyses of the role of mean graft flow in occlusion prediction per graft type

Variable	OR [95% CI]	Adjusted *P*-value
Arterial grafts		
* * ***MGF > 26.5 ml/min***	0.045 [0.005–0.44]*	0.008*
* *PI > 2	1.05 [0.170–6.5]	0.96
* *Target vessel size ≥ 1.75 mm	1.67 [0.260–10.7]	0.59
* *EuroSCORE-II (≥1.28 vs < 1.28)	0.244 [0.034–1.74]	0.16
Venous grafts		
* * ***MGF > 36.5 ml/min***	0.234 [0.055–0.99]*	0.049*
* *PI > 2	0.64 [0.115–3.5]	0.60
* *Target vessel size ≥ 1.75 mm	0.305 [0.035–2.69]	0.29
* *EuroSCORE-II (≥1.28 vs < 1.28)	2.93 [0.68–12.6]	0.15

We used the median values for target vessel size, PI and EuroSCORE-II. All the independent variables were used as dichotomous variables, with cut-offs selected based on appropriate ROC analyses as mentioned in the manuscript. * and bold italics are used to visually highlight the statistically significant results.

MGF: mean graft flow; OR: odd ratio; PI: pulsatility index; ROC: receiver operative characteristic.

## DISCUSSION

Graft patency is a strong predictor of short-/long-term survival and major adverse cardiac events [13]. Early graft occlusion can be due to a variety of technical factors (graft damage, positioning, kinking, twisting, purse string of the anastomosis or stretching, air bubble trap, plaque emboli or clots) causing insufficient or turbulent flow and graft thrombosis. Patients’ native biological characteristics (coronary artery size, vascular bed, collateral flow, gender or race) [14] may also play a contributing role. The long-term patency of venous grafts is further influenced by additional factors, such as quality of the vein conduit, local oxidative stress, overdistension and intraluminal turbulence leading to dilatation and intimal hyperplasia [15].

TTFM may facilitate the identification of intraoperative technical factors that can affect perioperative and early graft failure. Early graft occlusion rate following CABG is reported to be around 10% [16], and it is most frequently observed in vein grafts (15–20%) [17] compared to arterial (8%).

In our study, the routine use of intraoperative TTFM helped us to achieve a combined early graft failure of 5.2% (3% arterial; 8% venous). IMA grafts had a comparable occlusion rate (4% RIMA, <1% LIMA) to that reported in the literature [18]. The observed occlusion rates were influenced by the quality of the target vessel and the degree of proximal stenosis (<70%), as determined by the QCA and CT analysis.

We report a high early patency of endoscopically harvested venous conduits (92%), which is similar or better to the reported in the literature for either endoscopically or open technique harvested vein conduits. This finding supports current literature which suggests that endoscopic vein harvesting can give at least equivalent patency rate to open vein harvesting [3, 19] and also supports the results from recent randomized trials [20].

The CT angiographic subgroup analysis of the occluded grafts revealed that the majority (85.7% arterial; 40% venous) were performed on target native coronary arteries without significant proximal disease or anastomosed onto CTO arteries with well-developed collaterals. This supports previous reports which suggest that competitive flow from the native coronaries or aggressive collaterals and advanced diffuse disease of small re-canalized CTO arteries could play an important role in graft occlusion, especially for arterial grafts [21, 22].

Our results support a move towards systematic evaluation of the preoperative angiogram and/or associated intracoronary physiology data and more standardized surgical techniques including the routine use of TTFM to guide contemporary CABG revascularization strategies, especially when multiple arterial grafts are used [6, 23]. CT-angiography and conventional angiography with 3D-QCA are both considered equivalent in identifying proximal coronary artery stenosis [24] and this was proven to be the case in our analysis. However, the rising interest on functional assessment of the proximal coronary artery stenosis (fractional flow reserve > 0.80) can be used in addition to both conventional and CT-angiography.

In our analysis, we reported that MGF is predominantly influenced by the size of the target vessel ≥1.75 mm and sex, and it correlates with PI for both arterial and venous grafts, while there was no difference of patency rates or MGF when grafts are performed by a surgeon experienced in both, off- and on-pump techniques [25]. These results support previous findings showing reduced MGF for small coronaries [26], the negative impact of diffusely diseased coronary arteries [27] and the importance of adequate surgical expertise in different techniques.

We identified that grafts had lower MGF in females, with a median difference of 10 ml/min for arteries and 13.5 ml/min for veins which can be explained by the fact that the size of the coronary arteries and heart muscle is smaller in females and in accordance to similar findings reported by our group, in a different cohort of CABG patients [7].

The multivariable regression analysis in our study demonstrated that MGF was the most reliable and independent predictor of early graft failure, after adjusting for established risk factors. Although the PI was found to correlate inversely with MGF, it did not seem to provide further predictive information, a finding that goes against the already known and widely accepted, predictive value of the PI [8, 28] and it could only be explained by the small percentage of occluded grafts in the study.

In a retrospectively analysis of 345 patients, Lehnert reported a direct correlation between flows and graft patency, at 1-year follow-up using conventional coronary angiography. The authors reported an occlusion rate of 6.5% for IMAs and 16.9% for SVG. They recommended that satisfactory patency rates can be achieved when MGF is >20 ml/min for IMAs and >40 ml/min for SVG [13]. Di Giammarco *et al.* [28] proposed different cut-off targets for coronary territories with MGF <15 ml/min and a PI >3 for the left coronaries and PI >5 for the right coronary grafts. In a subgroup of only 44 patients from the randomized GRIIP-trial [29], the intraoperative use of TTFM failed to detect an advantage on predicting major adverse cardiac events and improved 1-year graft patency, probably due to a very small sample size and a significant loss in follow-up. A recent meta-analysis of TTFM studies recognized the clinical experience of the surgeon in the decision-making to revise an underperforming graft and PI >3, as good supporting markers for patent grafts [8].

Our study is reporting a very low graft failure rate with a predictive MGF cut-off value of 26.5 ml/min for arterial and 36.5 ml/min for venous grafts. However, the flow alone must be interpreted with caution and the decision to revise a graft (which in our cohort was 4.5%) should be in conjunction with the clinical, anatomical and physiological status of the patient at the time of the measurement. The routine use of intraoperative TTFM is recommended by the 2018 ESC/EACTS guidelines (Class IIa) and National Institute of Clinical Excellence (NICE) and its intraoperative use can influence the decision to revise up to 5% of poor-performing grafts during CABG, leading to better early patency rates [30].

Our study has a number of limitations. It is an observational study with retrospective analyses of collected data; however, this is partially mitigated by prospectively nature of data collection and having the CT-angiograms and 3D-QCA analysis performed by operators blinded to the TTFM measurements. Fractional flow reserve assessment of the proximal stenosis was not routinely performed which could have helped us to limit further the possibility of performing by-pass grafts to arteries with <70% proximal stenosis. We limited our outcome only on totally occluded grafts, thus we did not analyse data regarding partial or flow-limiting stenosis and we did not correlate our results with medium- or long-term postoperative clinical findings. Of all the arterial grafts in our cohort, radial artery conduits displayed a high occlusion rate, however, this should be interpreted with extreme caution given the very small number of radial artery used in this cohort (*n* = 13). The multivariable analysis is based on a small cohort of patients with low event rate and as such, the model is at risk of being over-fitted and further validation of the reported results, including the impact of gender on the MGF values, is required. This dataset is derived from a single-centre, single-surgeon experience, which may comprise a source of selection bias. However, our study is one of the largest that report on the use of TTFM to assess early graft failure with a CT angiogram and it is reporting a very low rate of early graft failure, reinforcing previous publications on the benefits that TTFM can offer to modern coronary artery by-pass surgery.

## CONCLUSION

MGF seems to be the most reliable predictor of early graft patency and is influenced by intrinsic biological factors but not by cardiopulmonary bypass or endoscopic vein harvesting. We demonstrate that MGF is lower in females and that it is mainly influenced by local haemodynamic factors such as vessel size ≥1.75 mm and PI. We estimate that MGF > 26.5 ml/min for arterial and MGF > 36.5 ml/min for venous grafts perform well in predicting early arterial and venous graft patency. Contemporary CABG is associated with very low early graft failure rates and TTFM is an effective tool of intraoperative quality assurance.

## SUPPLEMENTARY MATERIAL

Supplementary material is available at *ICVTS* online.

**Conflict of interest:** George Krasopoulos: receives honoraria for being a consultant, advisor and speaker for Medistim ASA and Abbott. Charalambos Antoniadis: founder, director and shareholder of Caristo diagnostic, a University of Oxford spinout company. 

### Data Availability Statement

All relevant data are within the manuscript and its supporting information files.

### Author contributions

**Andrea D'Alessio:** Conceptualization; Data curation; Methodology; Resources; Supervision; Visualization; Writing—original draft; Writing—review & editing. **Ioannis Akoumianakis:** Conceptualization; Data curation; Formal analysis; Methodology; Supervision; Writing—original draft; Writing—review & editing. **Andrew Kelion:** Conceptualization; Data curation; Investigation; Methodology; Supervision; Writing—review & editing. **Dimitrios Terentes-Printzios:** Conceptualization; Data curation; Formal analysis; Investigation; Supervision; Writing—review & editing. **Andrew Lucking:** Conceptualization; Investigation; Methodology; Supervision; Writing—review & editing. **Sheena Thomas:** Data curation; Investigation. **Danilo Verdichizzo:** Conceptualization; Resources; Supervision; Writing—review & editing. **Amar Keiralla:** Conceptualization; Resources. **Charalambos Antoniades:** Supervision; Validation. **George Krasopoulos:** Conceptualization; Data curation; Methodology; Supervision; Validation; Writing—original draft; Writing—review & editing.

### Reviewer information

Interactive CardioVascular and Thoracic Surgery thanks the anonymous reviewers for their contribution to the peer review process of this article.

## Supplementary Material

ivab298_Supplementary_Data

## ABBREVIATIONS

CABG: Coronary artery bypass graft
CT: Computed tomography
CTO: Chronic total occlusion
IMA: Internal mammary artery
MGF: Mean graft flow
PI: Pulsatility index
TTFM: Transit time flow measurement
