# Supplementary material for: Graft flow assessment and early coronary artery bypass graft failure: a computed tomography analysis
Source: Interact Cardiovasc Thorac Surg. 2021 Oct 27;34(6):974–81. doi: 10.1093/icvts/ivab298 (PMC9159422; doi:10.1093/icvts/ivab298)
Supplement: ivab298_Supplementary_Data [file ivab298_supplementary_data.zip › ivab298-suppl_data/Graftpatencymanuscript.ICVTS.Supplement.R2.doc]

**Supplementary Materials and Methods**

**Surgical procedures**

All patients underwent CABG by a surgeon experienced in both, on- and off-pump techniques. The majority of patients (74%) had their operation on-pump and 18% underwent concomitant valve surgery. On-pump cases were performed under normothermia and hematic-cold-cardioplegia. Hot shot was administrated to all those with concomitant valve cases. Off-pump CABG were performed with the aid of stabilizer (Getinge Acrobat-i), mister blower, intraluminal shunt and/or slings (Table 1). All patients had Aspirin 300mg within 3-5 hours from the operation and where on dual antiplatelet therapy of Aspirin 75mg and Clopidogrel 75mg daily thereafter, with maximum dose of Statin therapy, beta-blockers and angiotensin receptor inhibitors along with calcium channel blockers as indicated.

Internal mammary arteries (IMAs), saphenous veins (SVs) or radial arteries (RAs) were used as conduits. IMAs were harvested using skeletonized, extra-pleural technique while SVs were harvested using endoscopic technique (EVH, Vasoview, Hemopro-2, Maquet, Wayne, USA). The revascularization strategy was planned before the operation. The native coronary arteries were sized with the use of intra-arterial shunt or a probe. Patients with sequential or Y-grafts were excluded from this cohort.

**Graft patency assessment**

The patency of all grafts was assessed by coronary CT-angiography and interpreted blindly from TTFM measurements by an experienced cardiac CT-radiologist and/or imaging specialist cardiologist at a median of 11.4 weeks (range: 6-52 weeks) following CABG^1^. A graft was considered occluded when there was no opacification. All occluded grafts had the grafted native coronary artery along with the quality of the distal native coronary vascular bed evaluated, by an experienced imaging specialist cardiologist.

The preoperative angiograms of all occluded grafts were retrospectively assessed using QAngio XA 3-dimensional software package (Medis Medical Imaging System) by an interventional cardiologist blinded to the patency of the graft using standardized techniques^2^. All coronaries on occluded grafts were assessed for native vessel diameter and degree (diameter and area) of the stenosis. The CTO arteries with occluded grafts had the size of the distal myocardial bed filled by the target vessel categorized as small, medium, or large^3^. The extent of perfusion distal to CTOs through collateral feeding arteries was graded^4^ (0=no filling; 1=filling of side branches only; 2=partial epicardial vessel filling by collaterals and 3=complete epicardial vessel filling by collaterals).

**Supplementary Figures**

**Supplement Figure S1: Demographic determinants of graft flow.**

Mean graft flow (MGF) of both arterial and venous grafts was increased in male patients (A and B respectively). P-values are calculated by Mann Whitney U tests.

**Supplementary Tables**

**Supplemental table S1:** Graft occlusion rates per graft type

| **Graft type** | **Total graft number** | **Occluded graft number** | **Occlusion rate (%)** |
| --- | --- | --- | --- |
| Overall | 422 | 22 | 5.2 |
| SV | 187 | 15 | 8.0 |
| Arterial   - *LIMA* - *RIMA* - *RA* | 235  140  82  13 | 7  1  4  2 | 3  0.7  4.9  15.4 |

LIMA: Left internal mammary artery; RA: Radial artery; RIMA: Right internal mammary artery; SV: Saphenous vein;

**Supplemental Table S2: Descriptive graft characteristics per grafting site.**

| **Grafting site** | **Graft type** | | | | |
| --- | --- | --- | --- | --- | --- |
|  | ***SV***  *(n/occlusion)* | ***LIMA***  *(n/occlusion)* | ***RIMA***  *(n/occlusion)* | ***RA*** *(n/occlusion)* | ***Total*** *(n/occlusion)* |
| ***LAD***  ***Int***  ***Diagonal***  ***OM***  ***RCA***  ***PDA*** | 1/0  13/3  30/0  48/2  23/2  72/8 | 94/0  7/0  2/1  37/0  0  0 | 48/2  16/1  8/1  8/0  2/0  0 | 1/0  1/1  0  3/0  2/1  6/0 | 144/2 (1,3%)  37/5 (13.5%)  40/2 (5%)  96/2 (2%)  27/3 (11.1%)  78/8 (10.2%) |

Int: Intermediate artery; LAD: Left anterior descendent; LIMA: Left internal mammary artery; OM: Obtuse marginal; PDA: Posterior descendent artery; RA: Radial artery; RCA: Right coronary artery; RIMA: Right internal mammary artery; SV: Saphenous vein;

**Supplemental Table S3:** QCA and CT Sub analyses of the occluded grafts.

|  | **Pre-operative QCA**  n (%) | **Post-operative CT**  n (%) |
| --- | --- | --- |
| **Native artery stenosis <70%** | 13 (59,1%) | 12 (54.5%) |
| **CTO artery** | 5 (22,7%) | 6 (27.3%) |
| **Native artery stenosis >70%** | 4 (18,2%) | 4 (18.2%) |

CT: computerized tomography; CTO: chronically totally occluded; QCA: Quantitative coronary angiography

**Supplemental Table S4: 3D QCA characteristic of coronary stenosis and chronic occlusions.**

| **Anatomic characteristics coronary artery stenosis (n=17)** | |
| --- | --- |
| 3D-QCA RVD (mm) | 2.20 (1.90-2.65) |
| 3D-QCA diameter stenosis (%) | 53.00 (50.00-60.50) |
| 3D-QCA area stenosis (%) | 71.00 (65.00-81.00) |
| 3D-QCA MLD (mm) | 0.90 (0.70-1.20) |
| 3D-QCA MLA (mm^2^) | 0.90 (0.55-1.60) |
| CTFC (frames) | 19.41 (11.00-28.00) |
| **Chronic total occlusions (n=5)** | |
| Target vessel size and myocardial bed | |
| Small/Medium/Large, n (%) | 0 (0)/5 (100)/0 (0) |
| Grading of collaterals | |
| Grade 0/1/2/3; n (%) | 0 (0)/0 (0)/3 (60)/2 (40) |

Categorical variables are presented as absolute and relative frequencies, while continuous variables as median value (25th–75th percentile). 3D, three-dimensional; CTFC: Corrected TIMI frame count; LAD: Left anterior descending; LCx: Left circumflex; MLA: minimum luminal area; MLD: minimum luminal diameter; QCA: quantitative coronary angiography; RCA: Right coronary artery; RVD: Reference vessel diameter.

**Supplementary References**

1.	Leipsic J, Abbara S, Achenbach S, Cury R, Earls JP, Mancini GBJ et al. SCCT guidelines for the interpretation and reporting of coronary CT angiography: A report of the Society of Cardiovascular Computed Tomography Guidelines Committee. J Cardiovasc CT 2014;8(5): 342-58

2.	Pyxaras SA, Tu S, Barbato E, Barbati G, Di Serafino L, De Vroey F et al. Quantitative angiography and optical coherence tomography for the functional assessment of nonobstructive coronary stenoses: comparison with fractional flow reserve. *Am Heart J.* 2013;166(6):1010-1018.e1.

3.	Desai ND, Naylor CD, Kiss A, Cohen EA, Feder-Elituv R, Miwa S et al; Radial Artery Patency Study Investigators. Impact of patient and target-vessel characteristics on arterial and venous bypass graft patency: insight from a randomized trial. *Circulation.* 2007 Feb 13;115(6):684-91.

4.	Rentrop KP, Cohen M, Blanke H, Phillips RA. Changes in collateral channel filling immediately after controlled coronary artery occlusion by an angioplasty balloon in human subjects. *J Am Coll Cardiol.* 1985;5:587–92.
